# The Influence of Patient–Provider Communication on Cancer Screening

**DOI:** 10.1177/2374373520924993

**Published:** 2020-05-11

**Authors:** Tiffany B Kindratt, Folefac Atem, Florence J Dallo, Marlyn Allicock, Bijal A Balasubramanian

**Affiliations:** 1 Public Health Program, Department of Kinesiology, College of Nursing and Health Innovation, 12329 University of Texas at Arlington, Arlington, TX, USA; 2 Department of Biostatistics and Data Science, UT Health, School of Public Health Dallas, 12340 The University of Texas Health Science Center at Houston, Houston, TX, USA; 3 Department of Public and Environmental Wellness, School of Health Sciences, 6918 Oakland University, Rochester, MI, USA; 4 Department of Health Promotion and Behavioral Sciences, UT Health, School of Public Health Dallas, 12340 The University of Texas Health Science Center at Houston, Houston, TX, USA; 5 Center for Health Promotion and Prevention Research, UT Southwestern–Harold C. Simmons Comprehensive Cancer Center, Dallas, TX, USA; 6 Department of Epidemiology, Human Genetics, and Environmental Sciences, UT Health, School of Public Health Dallas, 12340 The University of Texas Health Science Center at Houston, Houston, TX, USA

**Keywords:** patient–provider communication, e-mail, cancer screening, Health Information National Trends Survey

## Abstract

Few studies have examined how different qualities and modes (face-to-face vs electronic) of patient–provider communication (PPC) influence cancer screening uptake. Our objective was to determine whether receiving a breast, cervical, and colorectal cancer screening is influenced by (1) qualities of face-to-face and (2) the use of e-mail PPC. We analyzed Health Information National Trends Survey 4, cycles 1 to 4 data. To assess qualities of face-to-face PPC, adults reported how often physicians spent enough time with them, explained so they understood, gave them a chance to ask questions, addressed feelings and emotions, involved them in decisions, confirmed understanding, and helped them with uncertainty. Adults reported whether they used e-mail PPC. We used multivariable logistic regression to evaluate the odds of receiving cancer screenings based on face-to-face and e-mail PPC. Adults whose health-care providers involved them in decision-making had highest odds of receiving breast (odds ratio [OR] = 1.38; 95% confidence interval [CI] = 1.11-1.71), cervical (OR = 1.30; 95% CI = 1.06-1.60), and colorectal (OR = 1.25; 95% CI = 1.03-1.51) cancer screenings. No significant associations were observed between e-mail PPC and cancer screenings. More research is needed to explore this association.

## Introduction

Despite the established benefits of routine screenings, national targets for receiving breast, cervical, and colon cancer screenings remain unmet (1). Effective patient–provider communication (PPC), both face-to-face during visits and electronically between visits, is important for shared decision-making, especially because of differences in recommended screening guidelines. This, in turn, can enhance screening uptake by patients. Although studies have begun to uncover how communication between providers and patients influence adults’ likelihood of receiving screenings, research is limited and findings are inconsistent on the roles that face-to-face and e-mail PPC play as enabling factors of preventive cancer screening uptake.

## Literature Review

### Face-to-Face PPC

The qualities of face-to-face PPC exhibited during clinical encounters may contribute to patients’ likelihood of receiving breast, cervical, and colorectal cancer screenings. In 2007, the National Cancer Institute developed a framework for patient-centered communication to improve face-to-face PPC across the cancer care spectrum (2). The framework specified that face-to-face PPC should encompass the following 6 qualities: (1) fostering healing relationships, (2) exchanging information, (3) responding to emotions, (4) managing uncertainty, (5) making decisions, and (6) enabling patient self-management. Recommended communication tasks differ across phases of the cancer continuum from prevention, screening, diagnosis, treatment, survivorship, and end of life (2). During the cancer screening phase, providers should provide linguistically accessible information about screening tests, encourage shared decision-making about screenings based on an assessment of risks and benefits, help patients navigate the system to obtain follow-up results, and address patients’ worries and concerns (2,3). Despite the development of this framework over a decade ago, national objectives for adults receiving breast, cervical, and colon cancer screenings remain unmet.

The evidence is mixed on whether adults’ perceptions of the qualities of face-to-face PPC increase their likelihood of receiving preventive services using nationally representative samples, specifically for cancer screenings. In a large nationally representative study of American adults, Villani and Mortensen found significant associations between women’s perceptions of the qualities of face-to-face PPC and receiving breast cancer screenings (4). However, in other studies, the qualities of effective face-to-face PPC were not associated with receiving breast cancer screenings (5,6). Few studies have been conducted to evaluate associations between women’s perceptions of the qualities of face-to-face PPC and receiving cervical cancer screenings. To our knowledge, only 2 studies have been conducted using nationally representative samples, and no significant associations were observed (4,6). Inconsistent results have been found in studies examining associations between adults’ perceptions of the qualities of face-to-face PPC and colon cancer screenings. Studies conducted by Carcaise-Edinboro and Bradley, Underhill and Kiviniemi, and Ho and colleagues found significant associations between adults’ perceptions of the qualities of face-to-face PPC and receiving colon cancer screenings (7–9). However, in other studies, the qualities of effective face-to-face PPC were not associated with receiving colorectal cancer screenings (4,10,11). Ling et al evaluated associations between the qualities of face-to-face PPC in the past 12 months and up-to-date colorectal screenings (fecal occult blood test [FOBT] in the past 12 months; sigmoidoscopy or colonoscopy in the past 10 years). They did not find any associations between adults’ perceptions of the qualities of face-to-face PPC and their likelihood of obtaining up-to-date colorectal cancer screenings (11).

Conflicting results in previous studies may be due to differences in methods for evaluating measures of face-to-face PPC and cancer screening time frames. Measures representing the qualities of face-to-face PPC variables have been evaluated based on actual survey responses (1 = always to 4 = never) (10,12,13), by reversing survey responses (1 = never to 4 = always) (8,13–20), as a dichotomous variable (always/usually vs sometimes/new or always/not always), as an ordinal variable (low, medium, or high face-to-face PPC), and a composite scale combining all measures from 0 to 100. Some studies evaluated colon cancer screenings “ever,” while others evaluated colon cancer screenings based on recommended intervals. The difference in measurement and analysis of specific aspects of PPC in previous studies makes it difficult to compare effects on cancer screening between them.

### E-Mail PPC

Advances in technology have allowed for PPC to extend beyond the traditional face-to-face encounter through electronic communications. In 2009, the Health Information Technology for Economic and Clinical Health Act supported the development of a national infrastructure for the implementation of electronic health records (EHRs) in clinical settings (21). The use of EHRs has allowed for direct modes of electronic communication, including communications by text messaging, mobile applications, video conferencing, social media, and e-mail (22). Increasing trends in the use of e-mail PPC have been observed since the early 2000s (23). Previous studies evaluating the role of e-mail PPC have identified its uses for asynchronous inquiries about nonacute issues, medication information, administration questions, and lab results (22). Furthermore, patients who used e-mail to communicate with their health-care provider reported that it allowed for more convenient access to communicate with their provider, an increased level of comfort when asking questions, and the ability to save messages (22). Many patients expressed an interest in communicating with their provider by e-mail, yet uptake remains low (22). Barriers included concerns about logistical issues and the inability to ensure the right person read their e-mails. For example, in an article by Couchman et al, over half of the patients stated that they had Internet access and were willing to use it for communicating with their health-care provider, yet only 5.8% reported e-mailing their provider (24). Despite limited use, studies have shown that e-mail PPC can show improvements in clinical outcomes among patients with diabetes and hypertension (25). Other studies found that e-mail PPC contributed to weight loss and medication adherence (26). Few studies have evaluated the associations between electronic forms of PPC and patients’ likelihood of receiving preventive service utilization. One of our preliminary studies found that adults who communicated with their health-care provider by e-mail had 1.51 times greater odds (95% confidence interval [CI] = 1.44-1.59) of receiving an influenza vaccine compared to those who did not use e-mail PPC (27). A study by Totzkay et al found that electronic medical record use, used as a platform for e-mail communication, was associated with receiving breast cancer screenings (28). Whether or not e-mail PPC can increase adults’ likelihood of receiving cancer screenings is yet to be determined.

To address the gap in our understanding of how PPC influences cancer screening uptake, we used a nationally representative sample to examine how 2 modes (qualities of face-to-face and e-mail) of PPC influence receipt of cancer screenings. Our aim is 2-fold: (1) examine associations between adults’ perceptions of qualities of face-to-face PPC and (2) their use of e-mail PPC and their likelihood of receiving breast, cervical, and colon cancer screenings. Our research questions are:

1. What qualities of face-to-face PPC are associated with increased odds of receiving breast, cervical, and colon cancer screenings?
2. Are adults who use e-mail to communicate with their health-care provider more likely to receive breast, cervical, and colon cancer screenings?

Our study evaluates qualities and modes of PPC as split components, which is similar to other frameworks such as the analytical hierarchy process for health technology assessment (29,30).

## Methodology

### Data Source

We analyzed cross-sectional data from Health Information National Trends Survey (HINTS) 4 (cycles 1-4). The HINTS 4 (2011-2014) was collected from adults by mail. Neighborhoods with high proportions of racial and ethnic groups were oversampled (31). Informed consent was obtained previously. Further details of the HINTS have been reported previously (32).

### Study Participants

We limited our sample to adults (aged ≥21 years) who reported a primary care visit in the last 12 months (n = 20 447) and no personal history of breast, cervical, and colon cancer for each screening of interest (n = 11 460). For breast cancer screenings, the sample included women aged 40 years or older (n = 4773) (33). For cervical cancer screenings, the sample included women aged 21 to 65 years (n = 4822) (34–36). For colon cancer screenings, the sample included adults aged 50 years and older (n = 5323) (34,37,38).

### Variables

#### Independent variables

Independent variables were adults’ perceptions of the qualities of face-to-face and e-mail PPC in the last 12 months.

#### Face-to-face PPC

In HINTS 4 cycles 1 to 4, adults who reported that they went “to a doctor, nurse, or other health professional to get care” for themselves at least once in the past year were asked 7 questions measuring the quality of the PPC interactions during their visits (32). Adults were asked how often their provider gave them a chance to ask questions, gave them the attention needed for feelings and emotions, involved them in decisions, confirmed their understanding of what was needed to take care of their health, explained so they could understand, spent enough time with them, and helped them deal with feelings of uncertainty. These qualities have been examined previously as valid measures of patient-centered communication (*r* = 0.76-0.79) (39). Adults rated each quality on a 4-point scale (always, usually, sometimes, or never). A dichotomous variable was created to compare providers who “always” versus “not always” (usually, sometimes, or other) exhibited each quality of face-to-face PPC based on previous studies (40–44).

#### E-mail PPC

Adults reported whether or not they “used e-mail to communicate with a doctor or doctor’s office” (HINTS 4 cycles 1 and 3) or “used e-mail to exchange medical information with a health-care professional” (HINTS 4 cycles 3 and 4) in the past 12 months (45). A dichotomous variable was created to compare adults who used e-mail to communicate and/or exchange health information with a health-care professional and those who did not.

#### Dependent variables

Dependent variables were breast, cervical, and colon cancer screenings. For breast cancer screenings, women were asked 2 questions to determine whether they *ever* received a mammogram, and if yes, how long ago it was received. For cervical cancer screenings, women were asked 2 questions to determine whether they *ever* received a Pap test, and if yes, how long ago it was received. Dichotomous variables (yes or no) were created to compare whether women received a mammogram or Pap test in the last 12 months. For colon cancer screenings, adults were asked “have you *ever* had a test to check for colon cancer?” (45). Responses were collapsed to create a dichotomous variable (yes or no) evaluating adults’ receipt of a colon cancer screening *ever*. Breast and cervical cancer screening variables were collected during all 4 cycles. Colon cancer screening variables were not collected during the fourth cycle.

#### Other covariates

We examined age, sex, race/ethnicity, nativity status, marital status, highest level of education achieved, insurance coverage, and perceived health status as potential covariates based on previous studies (4,7).

### Statistical Analysis

We used frequencies and percentages (unweighted and weighted) to describe sociodemographic characteristics and adults’ perceptions of qualities of face-to-face and e-mail PPC by receipt of breast, cervical, and colon cancer screenings. We used χ^2^ tests to evaluate differences for all variables. We calculated age-adjusted prevalence estimates of receiving breast, cervical, and colon cancer screenings among adults whose health-care providers “always” and “not always” exhibited specific qualities of face-to-face and e-mail PPC. We used logistic regression to examine associations between qualities of face-to-face and e-mail PPC (independent variables) and their likelihood of receiving cancer screenings before and after controlling for confounders. We used purposeful selection methods for model building (46). We used χ^2^ tests and Akaike information criterion to assess the goodness of fit (47).

### Sensitivity Analysis

We conducted sensitivity analyses to account for different age recommendations from national agencies and eliminate adults not due for screenings based on previous research (48). For breast cancer screenings, we conducted 2 sensitivity analyses. First, we limited the sample to women aged 45 years and older based on American Cancer Society (ACS) recommendations (34). Second, we limited the sample to women aged 50 years and older who did not receive a mammogram within 1 to 2 years ago based on United States Preventive Services Task Force (USPSTF) recommendations (49). For cervical cancer screenings, our sample was limited to women who did not receive a Pap test within 1 to 3 years ago (34–36). For colon cancer screenings, we limited our sample to adults’ ages 50 to 75 years based on USPSTF recommendations (38).

We used SAS 9.4 survey procedures for the data analysis of this complex survey design. We tested for the influence of missing data with χ^2^ and *t* tests. The HINTS weighting process included a final full-sample weight and 50 replicate weights for survey respondents in each cycle (32).

Our study was deemed exempt from human subjects review by the University of Texas Health Science Center at Houston’s Committee for the Protection of Human Subjects.

## Results

Table 1 displays sociodemographic characteristics of adults by receipt of breast, cervical, and colon cancer screenings using unweighted frequencies and weighted percentages. Results were similar for all cancer screenings. Women who reported having a breast cancer screening and adults who reported a colon cancer screening were older (aged 50-59 years), non-Hispanic white, born in the United States, married, have a bachelor’s degree or higher education, have health insurance coverage, and self-reported being excellent, very good, or good health. Women who reported having a cervical cancer screening in the last 12 months were slightly younger (aged 40-49 years), with negligible differences for other characteristics. More women (53.2%) received colon cancer screenings compared to men.

**Table 1.** Selected Characteristics by Cancer Screening in the Last 12 Months, n = 20 747.^a^

	Breast Cancer Screening (n = 5483)		Cervical Cancer Screening (n = 6842)		Colon Cancer Screening (n = 8422)^b^	
	No (n = 1785)	Yes (n = 3698)	No (n = 3243)	Yes (n = 3599)	No (n = 3807)	Yes (n = 4615)
Age						
21-29	35 (4.1)	37 (2.1)	134 (9.4)	400 (19.7)	549 (26.1)	33 (1.9)
30-39	139 (11.7)	128 (4.9)	292 (13.5)	648 (21.1)	918 (27.2)	118 (5.2)
40-49	290 (20.3)	708 (24.5)	409 (16.4)	764 (22.1)	1040 (25.3)	351 (11.5)
50-59	467 (23.8)	1053 (28.2)	687 (18.2)	871 (20.0)	668 (12.1)	1242 (29.0)
60-69	377 (15.5)	991 (21.6)	752 (17.6)	629 (11.2)	343 (4.8)	1533 (26.7)
≥70	477 (24.6)	781 (18.7)	969 (25.0)	287 (2.9)	289 (4.5)	1338 (25.7)
Race/ethnicity						
NH white	1039 (71.9)	2081 (70.2)	1958 (75.1)	1968 (65.2)	2180 (66.6)	2866 (74.8)
Hispanic	212 (11.2)	434 (11.5)	358 (10.4)	521 (13.8)	597 (16.2)	453 (9.9)
NH black	271 (12.3)	661 (12.4)	446 (9.9)	691 (14.6)	538 (10.9)	648 (10.1)
NH Asian/other	101 (4.6)	209 (5.9)	173 (4.5)	225 (6.5)	272 (6.4)	232 (5.2)
Foreign-born	236 (12.0)	460 (12.5)	350 (10.2)	523 (14.2)	617 (14.4)	484 (10.2)
Marital status						
Never married	228 (14.2)	416 (11.2)	408 (15.8)	661 (25.4)	823 (30.9)	468 (10.4)
Married/partner	721 (53.4)	1796 (63.7)	1381 (55.7)	1895 (60.0)	2018 (56.0)	2538 (66.2)
Other	814 (32.4)	1426 (25.1)	1411 (28.5)	994 (14.6)	912 (13.2)	1542 (23.4)
Education						
Less than HS graduate	189 (13.2)	323 (12.7)	330 (14.2)	256 (9.2)	337 (11.4)	415 (11.0)
HS graduate	413 (21.8)	807 (22.3)	746 (21.6)	648 (18.2)	718 (19.2)	995 (21.6)
Some college	566 (36.9)	1081 (30.8)	1029 (36.1)	994 (31.0)	1111 (35.0)	1362 (32.3)
Bachelor’s degree	604 (28.1)	1450 (34.2)	1108 (28.1)	1674 (41.6)	1605 (34.4)	1797 (35.1)
No health insurance	212 (14.6)	235 (8.3)	323 (15.0)	2906 (85.0)	506 (16.4)	203 (6.4)
Poor/fair health	428 (22.7)	580 (15.0)	686 (18.4)	2483 (81.6)	583 (13.8)	828 (16.6)

Abbreviations: HS, high school; NH, non-Hispanic; PPC, patient–provider communication.

^a^ All results *P* < .05 except race/ethnicity and nativity status for breast cancer screening and education for colon cancer screening. Sample frequencies (unweighted) and weighted percentages reported.

^b^ Only 3 cycles available for colon cancer screening.

Table 2 displays the qualities of face-to-face PPC and e-mail PPC by receipt of breast, cervical, and colon cancer screenings using unweighted frequencies and weighted percentages. Most adults who received a breast (65.5%), cervical (62.9%), and colon (66.3%) cancer screening reported that their health-care provider explained information in a way that they could understand. While over half of the adults who received breast and colon cancer screenings reported that their health-care provider spent enough time with them (52.9% and 53.5%, respectively), we found lower estimates among women who received a cervical cancer screening (45.9%). Most adults reported that their health-care providers always gave them a chance to ask questions (65.4% breast, 61.3% cervical, and 67% colon). However, fewer health-care providers involved adults in making decisions (58.7% breast, 54.3% cervical, and 58.5% colon). The use of e-mail PPC was low, ranging from 24% (breast) to 29.5% (cervical).

**Table 2.** Cancer Screening Estimates by Face-to-Face and E-mail PPC Characteristics, n = 11 460.^a^

Face-to-face PPC	Breast Cancer Screening (n = 4773)		Cervical Cancer Screening (n = 4972)		Colon Cancer Screening (n = 5323)^b^	
	No (n = 1512)	Yes (n = 3261)	No (n = 1923)	Yes (n = 3049)	No (n = 1298)	Yes (n = 4025)
Explained so understand						
Always	876 (61.3)	2084 (65.5)	1148 (58.1)	1935 (62.9)	749 (59.1)	2612 (66.3)
Spent enough time with you						
Always	706 (48.2)	1666 (52.9)	854 (44.8)	1430 (45.9)	584 (48.1)	2056 (53.5)
Understood next steps						
Always	913 (58.7)	2132 (63.0)	1085 (55.0)	1845 (60.0)	727 (57.5)	2558 (64.5)
Chance to ask questions						
Always	896 (60.5)	2131 (65.4)	1117 (55.8)	1882 (61.3)	741 (59.9)	2624 (67.0)
Addressed feelings						
Always	667 (46.0)	1651 (51.0)	813 (41.2)	1428 (46.3)	556 (44.1)	1972 (50.8)
Involved you in decisions						
Always	760 (51.6)	1859 (58.7)	954 (47.8)	1637 (54.3)	614 (50.8)	2279 (58.5)
Help with feelings of uncertainty						
Always	649 (44.2)	1544 (48.7)	777 (39.1)	1338 (43.4)	529 (42.8)	1871 (47.8)
E-mail PPC	202 (23.1)	514 (24.0)	392 (30.9)	583 (29.5)	110 (18.5)	507 (25.0)

Abbreviation: PPC, patient–provider communication.

^a^ All *P*s < .05 except explained so understand, understood next steps, and e-mail PPC for breast cancer screening, spent enough time, and helped with feelings of uncertainty for cervical cancer screening. Sample frequencies (unweighted) and weighted percentages reported.

^b^ Only 3 cycles available for colon cancer screening.

Table 3 displays the age-adjusted prevalence of receiving each cancer screenings by qualities of face-to-face and e-mail PPC. Age-adjusted prevalence estimates associated with the qualities of face-to-face PPC were highest for colon cancer screenings (∼78%) and lowest for cervical cancer screenings (ranged from 61% to 64%). Age-adjusted prevalence estimates for receiving a breast, cervical, breast, and colon cancer screening among adults who reported using e-mail PPC were 58%, 68%, and 84%, respectively.

**Table 3.** Age-Adjusted Prevalence of Screenings by PPC Measures.

Face-to-face PPC	Breast Cancer Screening: Age-Adjusted Prevalence (95% CI)	Cervical Cancer Screening: Age-Adjusted Prevalence (95% CI)	Colon Cancer Screening: Age-Adjusted Prevalence (95% CI)^a^
Explained so understood			
Not always	0.65 (0.62-0.69)	0.56 (0.53-0.60)	0.72 (0.68-0.75)
Always	0.70 (0.67-0.72)	0.62 (0.59-0.65)	0.77 (0.75-0.79)
Spent enough time with you			
Not always	0.66 (0.63-0.69)	0.59 (0.56-0.62)	0.74 (0.71-0.76)
Always	0.70 (0.68-0.73)	0.61 (0.58-0.65)	0.77 (0.74-0.79)
Understood next steps			
Not always	0.66 (0.62-0.68)	0.57 (0.53-0.60)	0.73 (0.69-0.76)
Always	0.70 (0.67-0.73)	0.63 (0.60-0.65)	0.77 (0.75-0.79)
Chance to ask questions			
Not always	0.65 (0.62-0.68)	0.56 (0.53-0.60)	0.72 (0.69-0.75)
Always	0.70 (0.68-0.73)	0.63 (0.60-0.66)	0.77 (0.75-0.79)
Addressed feelings			
Not always	0.66 (0.63-0.69)	0.58 (0.55-0.61)	0.74 (0.71-0.76)
Always	0.71 (0.68-0.74)	0.63 (0.60-0.67)	0.77 (0.75-0.79)
Involved you in decisions			
Not always	0.65 (0.61-0.68)	0.56 (0.53-0.60)	0.73 (0.69-0.76)
Always	0.71 (0.69-0.74)	0.64 (0.61-0.67)	0.78 (0.76-0.80)
Help with feelings of uncertainty			
Not always	0.66 (0.63-0.69)	0.58 (0.55-0.61)	0.74 (0.71-0.77)
Always	0.71 (0.68-0.73)	0.63 (0.60-0.66)	0.77 (0.74-0.79)
E-mail PPC			
No	0.69 (0.66-0.72)	0.60 (0.57-0.64)	0.76 (0.74-0.77)
Yes	0.68 (0.63-0.73)	0.58 (0.53-0.63)	0.84 (0.82-0.86)

Abbreviations: CI, confidence interval; PPC, patient–provider communication.

^a^ Only 3 cycles available for colon cancer screening.

### Logistic Regression Results

Table 4 displays results from the logistic regression analyses. To maintain consistency across screenings, we controlled for the confounding effects of age, race/ethnicity, marital status, highest level of education, insurance coverage, and perceived health status. We adjusted for sex in colon cancer screening models. In adjusted models, adults who reported involvement in their decision-making had higher odds of receiving breast (odds ratio [OR] = 1.38; 95% CI = 1.11-1.71), cervical (OR = 1.30; 95% CI = 1.06-1.60), and colon (OR = 1.25; 95% CI = 1.03-1.51) cancer screenings. We found similar results when we evaluated associations between adults’ chances to ask questions and all cancer screenings (breast OR = 1.28; 95% CI = 1.04-1.58; cervical OR = 1.27; 95% CI = 1.04-1.55; colon OR = 1.29; 95% CI = 1.05-1.57). Other qualities associated with increased odds of screening were helping deal with feelings of uncertainty (cervical OR = 1.23; 95% CI = 1.01-1.51), explaining things so that adults could understand (colon OR = 1.23; 95% CI = 1.01-1.53), spending enough time with adults (colon OR = 1.23; 95% CI = 1.01-1.52), and giving adults the attention needed for their feelings and emotions (colon OR = 1.28; 95% CI = 1.05-1.55).

**Table 4.** Adjusted^a^ Logistic Regression Models.

Face-to-face PPC	Breast Cancer Screening, OR (95% CI)	Cervical Cancer Screening, OR (95% CI)	Colon Cancer Screening,^b^ OR (95% CI)
Explained so understood			
Not always	1.00	1.00	1.00
Always	1.17 (0.94-1.45)	1.18 (0.95-1.47)	1.23 (1.01-1.53)
Spent enough time with you			
Not always	1.00	1.00	1.00
Always	1.15 (0.94-1.39)	1.08 (0.87-1.33)	1.23 (1.01-1.52)
Understood what needed to do			
Not always	1.00	1.00	1.00
Always	1.20 (0.96-1.49)	1.22 (0.99-1.49)	1.25 (1.01-1.53)
Chance to ask questions			
Not always	1.00	1.00	1.00
Always	1.28 (1.04-1.58)	1.27 (1.04-1.55)	1.29 (1.05-1.57)
Addressed feelings			
Not always	1.00	1.00	1.00
Always	1.21 (0.98-1.49)	1.21 (0.99-1.49)	1.28 (1.05-1.55)
Involved you in decisions			
Not always	1.00	1.00	1.00
Always	1.38 (1.11-1.71)	1.30 (1.06-1.60)	1.25 (1.03-1.51)
Help with feelings of uncertainty			
Not always	1.00	1.00	1.00
Always	1.21 (0.99-1.49)	1.23 (1.01 1.51)	1.21 (0.99-1.50)
E-mail PPC			
No	1.00	1.00	1.00
Yes	0.95 (0.74-1.22)	0.84 (0.64-1.10)	1.39 (0.99-1.95)

Abbreviations: CI, confidence intervals; OR, odds ratio; PPC, patient–provider communication.

^a^ All adjusted models accounted for age, race/ethnicity, marital status, highest level of education, insurance status, and perceived health status.

^b^ Only 3 cycles available for colon cancer screening.

Our sensitivity analyses results are presented in Supplementary Table 1 (breast), Supplementary Table 2 (cervical), and Supplementary Table 3 (colon). All CIs overlapped with our initial findings.

## Discussion

Our study revealed that involving adults in making decisions and giving them the chance to ask questions were the most influential qualities of PPC associated with adults’ likelihood of receiving cancer screenings. Furthermore, adults who used e-mail to communicate with their health-care provider were not more likely to receive breast, cervical, or colon cancer screenings. Three important findings were observed.

*First*, involving adults in making decisions and allowing them a chance to ask questions emerged as the most influential qualities of face-to-face PPC associated with receiving cancer screenings. Few other studies have examined these associations using nationally representative samples and have produced inconsistent results (5,50,51). Using a nationally representative sample from the Medical Expenditure Panel Survey (MEPS), Villani and Mortensen found that women whose health-care provider asked them to help make decisions about treatments had 29% greater odds of receiving a breast cancer screening over the past 1 to 2 years compared to those whose health-care provider did not always involve them in decisions about treatments (4). No significant associations were observed for cervical or colon cancer screenings. Underhill and Kiviniemi examined the likelihood of *adherent* and *ever* colon cancer screenings using original survey responses (never, sometimes, usually, and always) (8). They found that for every unit increase in involvement in decision-making, the odds of ever receiving a colon cancer screening were 1.17 times higher (95% CI = 1.03-1.33). Giving adults a chance to ask all the health-related questions that they had was also an influential quality associated with breast, cervical, and cancer screenings. To our knowledge, this is the first study to evaluate the influence of this quality of face-to-face PPC and adults’ likelihood of receiving cancer screenings using nationally representative samples.

*Second*, most individual qualities of face-to-face PPC evaluated were associated with colon cancer screenings (except help with feelings of uncertainty), whereas fewer qualities were associated with breast and cervical cancer screenings (only involvement in decision-making and chance to ask questions). Previous studies have used HINTS data to examine associations between whether or not health-care providers always listened carefully, explained things, showed respect, spent enough time with them, and involved patients in decisions and receiving *adherent* (11,10), *repeat* (10), and *ever* (10) receiving colon cancer screenings and produced mixed results. Underhill and Kiviniemi combined qualities of face-to-face PPC and found that the odds of any *adherent* and *ever* colon cancer screening were higher by 1.27 times (95% CI = 1.13-1.43) and 1.17 times (95% CI = 1.01-1.36), respectively, for every unit increase in the PPC quality scale (1 = poor to 4 = excellent) (8). Similarly, Ho and colleagues found that adults who reported high levels of PPC had 2.23 times higher odds (95% CI = 1.67-2.98) of colon cancer screenings *ever* (9). Our results may be different from previous studies due to the differences in measurement of face-to-face PPC and receipt of colon cancer screening. The first 2 iterations of the HINTS included face-to-face PPC questions based on the Consumer Assessment of Healthcare Providers and Systems supplement added to the MEPS in 2000 (52). The third iteration, HINTS 2007, included modifications to the face-to-face PPC questions and measures to assess *adherent* and *ever* receiving colorectal cancer screenings collectively and disaggregated for colonoscopy, FOBT, and sigmoidoscopy (8). In this study, we used data from HINTS 4 cycles 1 to 4 which were limited to measuring adults who *ever* received a colon cancer screening only.

*Third*, our study found that the use of e-mail PPC did not influence adults’ likelihood of receiving breast, cervical, and colon cancer screenings. Results indicated that adults had 39% higher odds of receiving a colon cancer screening yet were marginally not significant (95% CI = 0.99-1.95). To further explore this association, national surveys should measure the “quality” of different uses of e-mail PPC. The HINTS 4 asked whether adults used computers or the Internet to communicate with their health-care provider by e-mail during cycles 1 and 3. Adults’ interpretations of the reasons for e-mail PPC were not measured. Adults who responded “yes” may have interpreted automatic e-mails for appointment reminders or receiving diagnostic test results as e-mail PPC versus directly e-mailing their health-care provider about specific health concerns. During HINTS 4 cycles 3 and 4, this question was revised to directly assess whether adults exchanged health information with their health-care provider by e-mail. However, the reason for communication remained unmeasured by these surveys. Future iterations of the HINTS should be revised to fully capture communication behaviors to better determine the implications of e-mail PPC on cancer screenings.

### Strengths and Limitations

Our study had several strengths. We used nationally representative data to examine influences of face-to-face and e-mail PPC on adults’ likelihood of receiving 3 cancer screenings. Using a large nationally representative data source allowed us to examine several qualities of face-to-face PPC, e-mail PPC, and adjust for multiple confounders in our regression models. To address potential biases from missing data, we used a jackknife weighting process for determining estimates (53). A limitation of our study was that we examined adults’ likelihood of receiving cancer screenings using self-reported measures. While some studies have shown that self-reports are consistent with medical records using nationally representative and convenience samples, others have found that self-reported measures overestimate cancer screening estimates (54). Several concerns have been identified on overestimates of cancer screenings using national surveys due to social desirability, question wording, and the inability to determine the reason for receiving cancer screening tests (54). These concerns should be kept in mind when interpreting our findings.

## Conclusion

Our study demonstrates that several qualities of face-to-face PPC may influence adults’ likelihood of receiving cancer screenings. Adults whose health-care providers involved them in decision-making and gave them a chance to ask questions were more likely to receive cancer screenings. A common theme from our results is the importance of patient engagement. Adults who perceived that they were more engaged during appointments were more likely to receive cancer screenings. Efforts to increase adults’ engagement in cancer screening decisions can be made at the patient, health-care provider, systems, and population levels. Future studies are needed to explore how sociodemographic factors influence these results. To our knowledge, this is the first study to evaluate e-mail PPC and cancer screenings. As clinics encourage e-mail PPC, more research is needed to determine how best it can be incorporated into traditional health-care visits. As screening recommendations continue to change, and recommendations become more individualized (55), a comprehensive approach is needed to engage more adults in screening and ultimately reduce cancer morbidity and mortality.

## Supplemental Material

Supplemental Material, Supplementary_Tables_JPX - The Influence of Patient–Provider Communication on Cancer Screening Click here for additional data file. Supplemental Material, Supplementary_Tables_JPX for The Influence of Patient–Provider Communication on Cancer Screening by Tiffany B Kindratt, Folefac Atem, Florence J Dallo, Marlyn Allicock and Bijal A Balasubramanian in Journal of Patient Experience
