# Supplementary material for: The Influence of Patient–Provider Communication on Cancer Screening
Source: J Patient Exp. 2020 May 11;7(6):1648–57. doi: 10.1177/2374373520924993 (PMC7786660; doi:10.1177/2374373520924993)
Supplement: Supplemental Material, Supplementary_Tables_JPX - The Influence of Patient–Provider Communication on Cancer Screening [file Supplementary_Tables_JPX.pdf]

Supplementary Table 1. Sensitivity analysis for breast cancer screenings based on ACS (ages ≥45 years) and USPSTF (ages ≥50 years, no mammogram in past 1-2 years) recommendations.

	**Crude OR (95% CI)**	**Adjusted OR (95% CI)**	**Crude OR (95% CI)**	**Adjusted OR (95% CI)**
**Face-to-Face PPC**	**ACS Recommendations (≥45 years)**		**USPSTF Recommendations (ages >50 years)** ***No mammogram in past 1-2 years***	
Not Always (Ref)	1.00	1.00	1.00	1.00
Explained so understood	1.36 (1.11, 1.66)	1.30 (1.00, 1.69)	1.46 (1.18, 1.80)	1.44 (1.11, 1.87)
Spent enough time with you	1.29 (1.03, 1.61)	1.36 (1.04, 1.78)	1.39 (1.10, 1.75)	1.49 (1.12, 1.96)
Chance to ask questions	1.36 (1.10, 1.68)	1.50 (1.15, 1.96)	1.54 (1.24, 1.92)	1.74 (1.31, 2.31)
Addressed feelings	1.27 (1.02, 1.57)	1.36 (1.04, 1.77)	1.31 (1.05, 1.63)	1.41 (1.08, 1.86)
Involved you in decisions	1.45 (1.16, 1.80)	1.55 (1.19, 2.02)	1.58 (1.27, 1.98)	1.71 (1.30, 2.26)
Understood what needed to do	1.38 (1.11, 1.73)	1.41 (1.06, 1.87)	1.43 (1.14, 1.80)	1.49 (1.12, 1.99)
Help with Feelings of uncertainty	1.15 (0.92, 1.42)	1.21 (0.92, 1.59)	1.23 (0.97, 1.55)	1.34 (1.00, 1.80)
**E-mail PPC**				
No (Ref)	1.00	1.00	1.00	1.00
Yes	1.03 (0.79, 1.35)	0.92 (0.70, 1.21)	0.96 (0.72, 1.28)	0.86 (0.64, 1.15)

** All adjusted models accounted for age, race/ethnicity, marital status, highest level of education, insurance status and perceived health status unless otherwise stated. Abbreviations: ACS=American Cancer Society; CI=confidence interval; OR=odds ratio; PPC=patient-provider communication; ref=reference group; USPSTF=United States Preventive Services Task Force.*

Supplementary Table 2. Sensitivity analysis for cervical cancer screening restricted to women who did not receive a Pap test past 1-3 years.*

	Crude OR (95% CI)	Adjusted OR (95% CI)
**Face-to-Face PPC**		
Not Always (Ref)	1.00	1.00
Explained so understood	1.28 (0.96, 1.72)	1.36 (0.98, 1.90)
Spent enough time with you	1.01 (0.76, 1.33)	1.22 (0.89, 1.66)
Chance to ask questions	1.50 (1.14, 1.96)	1.74 (1.29, 2.37)
Addressed feelings	1.33 (1.02, 1.72)	1.49 (1.11, 2.00)
Involved you in decisions	1.23 (0.92, 1.63)	1.33 (0.97, 1.83)
Understood what needed to do	1.28 (0.98, 1.67)	1.37 (1.00, 1.87)
Help with Feelings of uncertainty	1.28 (0.96, 1.71)	1.54 (1.12, 2.11)
**E-mail PPC**		
No (Ref)	1.00	1.00
Yes	1.36 (0.91, 2.04)	1.13 (0.71, 1.79)

[*]All models adjusted for age, race/ethnicity, highest level of education, insurance coverage and perceived health status. Abbreviations: CI=confidence interval; OR=odds ratio; PPC=patient-provider communication; ref=reference group.

Supplementary Table 3. Sensitivity analysis for colon cancer screening restricted to adults ages 50 to 75 years based on USPSTF guidelines.*

	Crude OR (95% CI)	Adjusted OR (95% CI)
**Face-to-Face PPC**		
Not always (Ref)	1.00	1.00
Explained so understood	1.39 (1.12, 1.71)	1.26 (0.99, 1.60)
Spent enough time with you	1.27 (1.04, 1.54)	1.24 (0.99, 1.56)
Understood what needed to do	1.34 (1.09, 1.66)	1.27 (1.00, 1.61)
Chance to ask questions	1.33 (1.09, 1.62)	1.26 (1.01, 1.58)
Addressed feelings	1.31 (1.09, 1.58)	1.31 (1.05, 1.63)
Involved you in decisions	1.37 (1.14, 1.64)	1.26 (1.01, 1.55)
Help with Feelings of uncertainty	1.22 (1.01, 1.47)	1.24 (0.99, 1.56)
**E-mail PPC**		
No (Ref)	1.00	1.00
Yes	1.44 (1.03, 2.01)	1.31 (0.91, 1.88)

All adjusted models accounted for age, race/ethnicity, marital status, highest level of education, insurance status and perceived health status unless otherwise stated. Abbreviations: CI=confidence interval; OR=odds ratio; PPC=patient-provider communication; ref=reference group; USPSTF=United States Preventive Services Task Force.
